# Self-Perceived Social Support of Patients with Chronic Skin Diseases in Saudi Arabia: A Cross-Sectional Survey

**DOI:** 10.3390/jcm12165406

**Published:** 2023-08-20

**Authors:** Aesha Farheen Siddiqui

**Affiliations:** Department of Family and Community Medicine, College of Medicine, King Khalid University, Abha 61421, Saudi Arabia; afarheen@kku.edu.sa

**Keywords:** chronic skin disease, dermatological, social support, social health, Saudi Arabia

## Abstract

Background: Chronic skin diseases have been recognised as having a detrimental effect on patients’ social functions. Objectives: To assess the perceived social support in patients with chronic skin disease and its associated factors. Methods: A cross-sectional study was conducted between January and April 2019 on patients with skin diseases taking treatment at Asir Central Hospital, Abha, Saudi Arabia. Patients of both sexes aged above 18 years undergoing treatment for a skin disease in ACH for more than 3 months (chronic skin disease) were recruited by simple random sampling, and a total of 249 patients returned completed questionnaires. A self-administered questionnaire was used to collect relevant information about the background and disease characteristics of the patients and the Multidimensional Scale of Perceived Social Support (MSPSS). The software package IBM SPSS Statistics for Windows, Version 23.0. Armonk, NY, USA: IBM Corp., was used for data entry and analysis. Descriptive statistics were used for patient characteristics, and perceived social support was analysed according to specific scoring criteria. The Kruskal–Wallis test and Mann–Whitney U test were used to find out the association of background and disease variables with the perceived social support. Correlation analysis was used to find the relationship of social support with the age of the patient. All associations were considered significant at *p* < 0.05. Result: The mean age of the study group was 36.52 ± 14.22 years. The majority of the patients were females (71.1%). Atopic dermatitis was the most common skin disease reported, with 22.1% of all patients suffering from it. Mean scores of perceived social support score were low globally (24.97 ± 12.31), as well as in the three dimensions of significant other (7.75 ± 4.14), friends (7.90 ± 4.59), and family support (9.40 ± 5.48). A significant difference in social support was perceived by patients with a disease duration of 3 months to 1 year and those with papulo-squamous skin disease as compared to acne. The presence of skin disease in a second-degree relative indicated a higher level of social support perception and had a significant positive correlation (r = 0.194, *p* = 0.002) with the age of the patient. Conclusion: Saudi patients with chronic skin disease have low social support. Some important insights into the functioning of social support were suggested by the study finding, which pointed to the significant effect of disease duration, type of disease, and presence of disease in second-degree relatives on the perception of social support in patients with a variety of chronic skin diseases. Qualitative exploratory and prospective research could help in understanding this aspect of psychosocial health in a better way and help to provide solutions.

## 1. Introduction

The skin serves many functions, besides having an aesthetic role [1]. Skin diseases are fairly common, with up to 14% of all consultations in general practice representing a skin disease [2]. The burden of skin disease is added to by the psychological impact of visible skin lesions, and the patients have not only to cope with the effects of their disease but also the reaction of others to their condition [3]. There is stigma attached to a wide range of skin diseases, affecting many millions of people, just as there is for mental illness and sexually transmitted infections [3].

Usually, chronic skin conditions are not curable; however, they are managed using drugs and by modifying one’s lifestyle [4,5,6]. Chronic pruritis in atopic dermatitis; painful fissures seen in severe eczema; burning sensations reported in psoriasis; intractable itching in pemphigus vulgaris; and inflammatory, painful, deep-rooted lesions in hidradenitis suppurativa have severe effects on patients’ wellbeing [7,8,9], and side effects from treatments such as corticosteroids, dupilumab, and adalimumab can have a bad effect on patients’ overall appearance [5,8,9,10,11]. They can even have a significant impact on personal relationships [12]. Skin diseases can be debilitating to patients in many ways. When treating patients with skin diseases, more focus should be put on psychosocial interventions. Existing research has shown the value of psychosocial treatments such as structured educational training, in-person and online cognitive behavioural therapy, and mindfulness-based cognitive therapy. These interventions are helpful in encouraging adaptation and assisting patients cope with their condition in a positive way, which improves patient outcomes [13].

Many studies have addressed the physical, psychological, and social functioning of patients with skin diseases [1,2]. Psychosocial factors such as mental health, quality of life, self-esteem, self-efficacy, resilience, social support, and social connection contribute to the adaptation to living with a chronic disease [14]. Among these factors, the role of social support is outlined as care, help, and support perceived as available for an individual when adapting to a life with chronic disease [15]. This study measured three components of psychosocial health in patients with chronic skin disease, namely, quality of life, mental health, and perceived social support, as well as the relationship between these variables. This paper presents findings about the perceived social support component, and the mental health and quality of life components will be presented in separate articles. 

Although different social support categories have been identified, five categories of social support: instrumental, informational, tangible, emotional, and spiritual, are relevant in the context of disease adaptation [14]. Instrumental support entails giving guidance and using other covert techniques to help the person handle a stressful situation more successfully. These could, for instance, be guidelines on how to handle a specific issue or act in a particular circumstance. Informational support is defined as giving a patient information that is pertinent to their illness or health. Such knowledge can be acquired from professional (such as physicians, nurses, and the scientific literature) or non-professional sources (such as other patients, superstitions), and it aids the patient in understanding their health-related circumstance [16]. Obtaining tangible support entails securing material aid, such as financial assistance or other required goods. Emotional support entails arousing in the patient the feelings and emotional states that can lessen the patient’s negative effects and, in turn, make it easier for them to cope emotionally with the illness. The actions of others (such as a minister or prayer group) that assist the patient in attributing a more profound, spiritual significance to their illness or pain are referred to as spiritual support. Globally speaking, therefore, disease-related social support is the sum of the perceived emotional and spiritual support, material and instrumental assistance, and information that is offered to a person when dealing with the disease [14].

In the recent past, there has been an increasing focus on the role of social support as a coping mechanism in patients with chronic disease [13,14]. Social support has been defined as an exchange of relationships or a transaction between individuals, which is believed by the provider of social support and/or the recipient to have a positive effect. Social support is provided by the community, family, or a close relation. It is described in various dimensions, such as directionality, content, or networks of social support. A number of scales have been designed to measure social support. Quantitative measures are designed to evaluate the number of social relationships and frequency of support incidents, while qualitative measures explore the perceptions of availability, adequacy, or value of the social support. Thus, there is a strong subjective dimension to this construct. 

A review of the relevant literature showed that social support is vitally associated with the way a patient handles the disease-related physical and psychological changes that occur to their body [17]. People who receive support from their family, friends, or significant others are healthier and cope more easily with the difficulties caused by an illness. This seems to be significant for visually expressed disease such as skin disease; however, as far we know, it has not been adequately addressed in Saudi Arabia [18]. In order to adapt to the disease; affected individuals need to develop psychological mechanisms along with social support in the form of family and friend support. This has been substantiated by many studies that reported on factors that affect adaptation to living with a chronic skin disease, emphasising social support to be strongly involved in the process of adaptation [14].

From a clinical perspective, the issue is worthy of study because it may produce results that can be helpful in the management of dermatology patients. In Saudi Arabia, health services are delivered in both government and private sector [19]. All government sector services are free of cost for Saudi citizens, and patients with skin disease avail medical services at both primary and higher levels of care. Primary healthcare centres (PHCCs) in Saudi Arabia are the first contact between the dermatology patients and the healthcare system, where either general practitioners or family physicians cater to these patients, and, if needed, refer them to specialist services at the secondary and tertiary level hospitals [20]. In the private sector, dermatology clinics are available in most large cities, with the availability of paid specialists and superspecialist services. Thus, our research aims to analyse perceptions about social support in patients with chronic skin diseases under treatment in a tertiary care centre in Asir, Saudi Arabia, and find its associated factors. 

## 2. Methodology

This study employed a cross-sectional design. The study was conducted between January and April 2019. The target population was chosen as patients with a chronic skin disease, and the sampling frame was the patients with skin diseases taking treatment at Asir Central Hospital, Abha. The Raosoft sample size calculator, available online, was used to estimate the sample size. To calculate the sample size, a comparative figure was needed; however, in the literature review, there was no local or regional study available for comparison, and consequently, to use the formula for calculating sample size, it was assumed that 50% of patients have poor social support. A 95% confidence interval and a 5% margin of error were applied, and the resultant target sample size was calculated to be 377. The required number of patients was selected by a simple random sampling method from the dermatology OPD of Asir Central Hospital. A total of 300 patients were identified who met the study criteria. The inclusion criteria encompassed all patients of age 18 years and above, of male or female sex undergoing treatment for a skin disease in ACH for more than 3 months (chronic skin disease). Child patients and those with a treatment window of less than 3 months were excluded. Consecutive sampling was used until the required sample size was reached. Out of 300 patients, 249 patients returned all completed questionnaires and were included as the final sample. Incomplete and incorrectly filled questionnaires were excluded. 

A self-administered questionnaire was used for collecting the data. The questionnaire consisted of two parts. Part one included questions on basic background and disease characteristics of the patients. The background variables that were included for the study purpose were age, sex, education, and residence. The disease characteristics included clinical diagnosis, disease duration, and treatment duration. 

The second part of the questionnaire consisted of various scales for assessing the psychosocial health, quality of life, and perceived social support. For mental health, the Depression, Anxiety and Stress Scale (DASS) was used [21]. The patient’s quality of life was assessed using the Dermatological Life Quality Index (DLQI) [22]. The third component that is perceived social support has been described in detail in this paper and was measured using the Multidimensional Scale of Perceived Social Support (MSPSS)—Arabic version [17]. The Multidimensional Scale of Perceived Social Support (MSPSS) has been widely used for some decades across cultures and languages [17,18]. It was developed by Zimet, Dalhem, Zimet, and Farley in 1988 [23], and it aims to measure the subjective dimension of social support from three specific sources, namely, family, friends, and significant others. This self-administered scale includes 12 items with 4 items for each dimension, namely, family, friends, and significant others. Support from a significant other may be interpreted variously to mean a particularly close relationship. Each of the twelve items is rated on a seven-point Likert-type scale ranging from 1 (very strongly disagree) to 7 (very strongly agree). The total score, calculated by summing the results for all items, is used to interpret the perception of social support. The score can possibly range from the lowest score of 12 to a highest of 84, with the higher score indicating higher perceived social support. In addition, support in the three subscales can be observed by calculating the score from the responses in each of the three dimensions on a scale of 4 to 28. The MSPSS instrument is short, with a reading level of comprehension of the third grade. Its validity and reliability has been established across different languages and cultures [17,18].

Data were entered into and analysed using Statistical Package for Social Sciences software (IBM SPSS Statistics for Windows, Version 23.0. Armonk, NY, USA: IBM Corp). Data were coded and entered into SPSS, specifying variable names. Initial analysis was conducted to find the frequency and percentages of the descriptive data for the study population, that is, the patient characteristics and disease characteristics. All basic characteristics are presented as frequency tables. To analyse the perceived social support, specific scoring criteria as described in the MPSS scale was used. The three dimensions of social support that were analysed were the dimensions of significant other, family and friends, and global perceived social support. The Kolmogorov–Smirnov goodness of fit test and Shapiro–Wilk test confirmed a non-normal distribution of the MPSS score, and thus the non-parametric tests of significance such as the Kruskal–Wallis and Mann–Whitney U test were used to find out the association of various variables with the perceived social support scores. Correlation analysis was used to determine the relationship of social support with age of the patient. In all cases, a *p*-value of ≤0.05 was considered as statistically significant. The study was conducted consequent to approval by the institutional ethics committee (King Khalid University Research Ethics Committee and Asir Central Hospital Ethics and IRB (Internal Review Board) Committee), approval number 2019-01-14. The patients were briefed about the objective of the study, and their consent was taken for participation. The study was entirely self-financed.

## 3. Result

Table 1 describes the background characteristics of the study group. The mean age of the study group was 36.52 ± 14.22 years. The majority of the patients were less than fifty years of age, with 40% being in the less than thirty years age group. The gender distribution showed that the majority of patients were females (71.1%). The educational level of the study group showed that 61% had acquired college education, and 21.3% had high school education. Fifty seven percent of the study group was married, while 36.9% was single. Almost three-quarters (72.3%) of the study group lived in cities, and 27.3% were living in villages. 

Table 2 reveals the pattern of skin diseases in the study group. Hypersensitivity disorders, including atopic dermatitis, were the most common skin diseases, with (29.7%) of all respondents suffering from them. The other common disorders were pigmentation disorders (19.3%), acne (18.9%), and papulo-squamous disorders including psoriasis (12.4%).The disease duration varied across the study group almost evenly. More than half (52.6%) were suffering from skin disease for 3 months to one year. Treatment duration of 3 months to one year was reported by 91.2% of the patients, 1–3 years treatment duration was reported by 3.2%, and 5.6% had been continuing treatment for more than 3 years. Similar disease in the family was reported by more than half (55%) of the patients, of which most reported it in first-degree relatives.

The preliminary findings of the mental health component (DASS) and the quality-of-life component (DLQI) are presented in Table 3. All three components of mental health showed a low score, indicating low levels of stress, anxiety, and depression in the study group. For quality of life, DLQI scores from 6 to 10 represent a moderate effect on quality of life, while scores above 10 represent a very large or extreme effect on quality of life. In the study group, the mean DLQI score was 6.45 ± 4.29, which indicates a moderate effect on the patient’s life quality. These findings are presented here for providing context to the reader, and the detailed results will be published separately.

Table 4 presents the descriptive statistics of the Multidimensional Scale of Perceived Social Support score in the study group. In this scale, the minimum possible global score was 12, and the maximum possible score was 84. The mean global MSPSS score was 24.97 ± 12.31. The mean score in the three dimensions, that is, significant others, friends, and family, were low (7.75 ± 4.14, 7.90 ± 4.59, and 9.40 ± 5.48, respectively).

While no significant differences in perceived social support across the three dimensions were observed based on the socio-demographic factors except age, differences in mean scores were found to exist based on disease-related factors. Spearman’s rho was used to study the correlation of age with MPSS score, and it was found to be significantly positively related (r = 0.194, *p* = 0.002), suggesting that social support was perceived to be higher with increasing age. Post hoc analysis by the Dunn–Bonferroni method revealed that significantly low global MPSS score was attained in those patients with papulo-squamous skin disease as compared to acne (*p* < 0.05). There were significantly high scores of family social support in those with a second-degree relative with skin disease (*p* < 0.05). In patients with disease duration between 3 months and 1 year, significantly low scores of social support in the significant other, family, and global dimension were observed(*p* < 0.05). This information is presented in Table 5.

## 4. Discussion

Social support is an important construct in psychosocial health. In difficult life circumstances, social support helps to placate the person [14,15] and is strongly implicated in the process of adaptation to disease [24]. Although social factors have been reported as particularly essential in adaptation to disease, it is conspicuous that a small number of studies have investigated the role of social support in patients with skin diseases [25]. Considering this research gap, this study was undertaken, and it is the first study in this region to address this issue.

The study population was composed of a relatively young age group, with a majority of the patients being less than fifty years of age. Most of the study group comprised college-educated, female patients, with the majority living in urban areas. Hypersensitivity disorders including atopic dermatitis were the most common skin diseases. The other common disorders were pigmentation disorders, acne, and papulo-squamous disorders including psoriasis. The disease duration varied across the study group almost evenly. More than half of the study group were suffering from skin disease for 3 months to one year, as also the treatment duration of 3 months to one year was reported by the majority of the patients. An important aspect of disease characteristics we studied was the presence of a similar condition in the family. More than half of the patients reported similar disease in the family, mostly in first-degree relatives. This basic information of the socio-demographic characteristics and disease distribution in the patients will help to reflect on the study findings about the perceptions of social support from significant others, family, and friends.

In this study, the perception of social support by patients with chronic skin disease was poor. The overall (global) mean score of perceived social support as well as mean score in all three dimensions, i.e., significant other, family, and friends, was found to be low. Social support is a process that arises through interactions between people. The interactions and relationships that shape social support formulate perceptions of and expectations about those interactions [14]. Perceived social support, especially support from a significant other, might help explain the complexities of the relationship between social support and health. Relationships that provide a feeling of security, such as parents or spouse, might be more effective in buffering stress [15]. Additionally, a secure relationship is likely associated with a greater willingness to seek support with the belief that support would be available and effective if needed [15,24]. Previous studies have shown that a social phenomenon such as poor social support exerts a negative influence on psychosocial functioning in patients with skin diseases [26,27]. A recent study from Greece concluded that psychosocial factors such as patients’ social support influence their psychological health and the way they experienced their skin condition, before treatment as well as in the follow-up period [28]. Considering these factors, the study findings hold implication for the treatment outcomes and overall wellbeing of the patients.

In this study, no significant differences in perceived social support across the three dimensions were observed based on gender, income, or residence; however, significant differences in mean scores were found to exist based on some of the disease-related factors. Previously, some studies have reported gender-based differences in perceived social support [29,30,31]; however, other studies [13] showed that the women and men with chronic skin disease reported similar levels of perceived social support. This was corroborated by the findings in our study. Generally, women report better social networks and resulting support, and in closed societies such as Middle Eastern countries, this may be strongly exhibited. In the current study, patients with a second-degree relative having skin disease showed significantly higher scores of family social support as compared to those with first-degree relatives. This finding is interesting, as it emphasises how social networks are important in determining psychological well-being of patients with stigmatising diseases [32,33]. Perception of social support depends on the recipient and, coupled with the size of the social support network, it influences health [34].

In patients with disease duration between 3 months and 1 year, significantly low scores of social support from significant others, family, and the global dimension were observed. Chronicity complicates the psychosocial dimensions of disease and affects the social support–health relationship. Social support has been considered a major component of self-management for chronic diseases and is associated with improved outcomes [5,34,35]. This finding is important as it may help the clinicians to tailor the social support needs of their patients during the different phases of treatment.

There was a significantly low global MPSS score attained in those patients with papulo-squamous skin disease as compared to acne. These findings are in concordance with previous research. To understand these findings, it is important to project the clinical picture of psoriasis here. Psoriasis lesions are thick, red, and scaly, and they may appear on any part of the body. They are often accompanied with pain and itching. These characteristics expose psoriasis patients to significant psychological distress and psychiatric morbidity. Many studies have reported experiences of stigmatisation and decreased health-related quality of life in patients with psoriasis. A study from Norway showed that patients with psoriasis scored significantly higher than healthy controls concerning skin-related shame and disgust, along with feelings of stigmatisation because of their visible lesions coupled with feelings of social exclusion and devaluation [36,37]. Recently published reviews have reported that perceived social support is positively correlated to scores on health measures, which indicated higher functioning in patients with acne and eczema [38,39]. Though some studies have shown patients with acne to have levels of social, psychological, and emotional impairments similar to those of serious diseases [40], in the current study, acne did not have a significant difference in social support from other dermatological disorders. This may be due to the difference in the age group of this study subjects, as acne is known to affect adolescent age groups more commonly [3,28].

## 5. Conclusions and Recommendations

There have been huge strides in the management of skin diseases to ease their burden. However, the psychosocial dimension of chronic skin disease management has remained largely ignored [41]. This study is a step in the direction to provide baseline information about the existing perception about social support in patients with chronic skin disease in the Asir region of Saudi Arabia. Studies in other parts of the world have shown that improvement of social support through psychotherapeutic interventions may benefit patients with chronic skin diseases for better social adjustment [36]. Thus, taking a cue from the findings of this study, dermatologists and public health experts in the region need to develop a stronger database on the psychosocial aspects of chronic skin diseases, as well as identify and address focal issues related to psychosocial health of the patients. Performing longitudinal prospective randomised control studies and qualitative studies may provide further evidence. It is hoped that the findings of this study can serve to motivate health authorities to prioritise the integration of psychosocial health with therapeutic treatment of patients with chronic skin conditions.

## 6. Strengths and Limitations

This study is a first study in the region describing perceived social support by patients with chronic skin diseases and identifying significant factors associated with them. However, the researcher realises that there are several limitations of this work. Firstly, this is a single-centre, cross-sectional study, and thus it is not possible to draw any conclusions regarding causality and directionality. The results are not generalisable to other populations. Another factor that limits the study findings was the presence of a variety of skin disorders, with varying clinical patterns and outcomes that were included in a single study. Although this provided important comparisons, it will be more useful if larger samples of each different skin disease were studied individually. Skin diseases such as acne have different implications for adolescents, while other conditions such as hyper-pigmentation may affect females at different stages of their life. It would be ideal to conduct qualitative research by individual interviews to assess social support, rather than the perception of social support. Thus, it is necessary to address these issues in more detail in future studies.

## Figures and Tables

**Table 1:** Basic characteristics of the study group.

Characteristic		Frequency	Percentage
Age group	18–30 years	103	41.4
	30–50 years	110	44.2
	More than 50 years	36	14.5
Sex	Female	177	71.1
	Male	72	28.9
Educational level	No formal education	19	7.6
	Elementary school	17	6.8
	Intermediate school	8	3.2
	High school	53	21.3
	Bachelor’s degree	152	61.0
Marital status	Divorced	13	5.2
	Married	142	57.0
	Single	92	36.9
	Widow	2	0.8
Residence	Urban	181	72.7
	Rural	68	27.3

**Table 2:** Disease characteristics in the study population.

Disease Characteristic	Frequency	Percent
Disease		
Atopic dermatitis and hypersensitivity diseases	74	29.8
Pigmentation disorders	48	19.3
Acne	47	18.9
Papulosquamous disorders (including psoriasis)	31	12.4
Skin infections	27	10.8
Others	22	8.8
Disease duration		
3 months–1 year	131	52.6
1–3 years	75	30.1
>3 years	43	17.3
Treatment duration		
3 months–1 year	227	91.2
1 year–3 years	8	3.2
>3 years	14	5.6
Similar disease in relative		
None	112	45.0
First-degree relatives	127	51.0
Second-degree relatives	10	4.0

**Table 3:** Mean scores of mental health and quality of life in the study participants.

Depression	Stress	Anxiety	Quality of Life
3.83 ± 3.29	4.90 ± 3.59	5.48 ± 4.26	6.45 ± 4.29

**Table 4:** Description of the global and dimensional perceived social support score.

Descriptive Statistics	Multidimensional Perceived Social Support Score			
	Significant Other	Family	Friends	Global Score
Mean ± std. deviation	7.75 ± 4.14	7.90 ± 4.59	9.40 ± 5.48	24.97 ± 12.31
Std. error of mean	0.26	0.29	0.34	0.78
CI 95% lower bound	7.23	7.32	8.72	23.43
Upper bound	8.27	8.47	10.09	26.50
Median	6.00	7.00	8.00	23.00
Minimum	4.00	4.00	4.00	12.00
Maximum	20.00	28.00	28.00	72.00
Range	16.00	24.00	24.00	60.00
Interquartile range	5	6	7	17
25th percentile	4.00	4.00	5.00	14.50
50th percentile	6.00	7.00	8.00	23.00
75th percentile	9.00	10.00	12.00	31.50

**Table 5:** Background and disease-related differences between mean scores of multidimensional perceived social support scale (MPSSS).

Patient Characteristics	Significant Other		Family		Friends		Global Score	
	k/U	Sig	k/U	Sig	k/U	Sig	k/U	Sig
Sex	0.50	0.61	−0.32	0.74	−0.906	0.36	−0.44	0.65
Marital status	2.96	0.39	0.34	0.95	0.663	0.88	0.06	0.99
Urban/rural	0.93	0.35	1.79	0.07	0.56	0.57	0.89	0.36
Education level	2.99	0.55	3.91	0.41	1.52	0.82	2.51	0.64
Skin condition	4.15	0.38	5.02	0.28	7.97	0.09	9.56	0.04 *
Family history	4.09	0.25	3.99	0.26	9.27	0.02 *	6.41	0.09
Duration	8.89	0.01 *	13.20	0.01 *	5.12	0.07	8.62	0.01 *

k = Kruskal–Wallis statistic; U = Mann–Whitney U statistic, * *p* < 0.05.

## Data Availability

The data presented in this study are available on request from the corresponding author.

## References

[1] Shiva Shanker Reddy M., Chaturvedi S.K. (2017). Psychosocial Issues in Dermatology. EMJ Dermatol..

[2] Hazarika N., Archana M. (2016). The psychosocial impact of acne vulgaris. Indian J. Dermatol..

[3] Dunn L.K., O’Neill J.L., Feldman S.R. (2011). Acne in adolescents: Quality of life, self-esteem, mood, and psychological disorders. Dermatol. Online J..

[4] Green L. (2010). The effect of skin conditions on patients’ quality of life. Nurs. Stand..

[5] Mastorino L., Rosset F., Gelato F., Ortoncelli M., Cavaliere G., Quaglino P., Ribero S. (2022). Chronic Pruritus in Atopic Patients Treated with Dupilumab: Real Life Response and Related Parameters in 354 Patients. Pharmaceuticals.

[6] Martora F., Marasca C., Battista T., Fabbrocini G., Ruggiero A. (2022). Management of patients with hidradenitis suppurativa during COVID-19 vaccination: An experience from southern Italy. Comment on: ‘Evaluating the safety and efficacy of COVID-19 vaccination in patients with hidradenitis suppurativa’. Clin. Exp. Dermatol..

[7] Martora F., Scalvenzi M., Ruggiero A., Potestio L., Battista T., Megna M. (2023). Hidradenitis Suppurativa and JAK Inhibitors: A Review of the Published Literature. Medicina.

[8] Miniotti M., Ribero S., Mastorino L., Ortoncelli M., Gelato F., Bailon M., Trioni J., Stefan B., Quaglino P., Leombruni P. (2023). Long-term psychological outcome of patients with moderate-to-severe atopic dermatitis continuously treated with Dupilumab: Data up to 3 years. Exp. Dermatol..

[9] Martora F., Martora L., Fabbrocini G., Marasca C. (2022). A Case of Pemphigus Vulgaris and Hidradenitis Suppurativa: May Systemic Steroids Be Considered in the Standard Management of Hidradenitis Suppurativa?. Skin Appendage Disord..

[10] Mastorino L., Viola R., Panzone M., Avallone G., Gallo G., Ortoncelli M., Cavaliere G., Quaglino P., Ribero S. (2021). Dupilumab induces a rapid decrease of pruritus in adolescents: A pilot real-life study. Dermatol. Ther..

[11] Martora F., Marasca C., Fabbrocini G., Ruggiero A. (2022). Strategies adopted in a southern Italian referral centre to reduce adalimumab discontinuation: Comment on ‘Can we increase the drug survival time of biologic therapies in hidradenitis suppurativa?’. Clin. Exp. Dermatol..

[12] Yarbrough K.B., Neuhaus K.J., Simpson E.L. (2013). The effects of treatment on itch in atopic dermatitis: The effects of treatment on itch in AD. Dermatol. Ther..

[13] Janowski K., Steuden S., Pietrzak A., Krasowska D., Kaczmarek Ł., Gradus I., Chodorowska G. (2012). Social support and adaptation to the disease in men and women with psoriasis. Arch. Dermatol. Res..

[14] Fasihi Harandi T., Mohammad Taghinasab M., Dehghan Nayeri T. (2017). The correlation of social support with mental health: A meta-analysis. Electron. Phys..

[15] Strom J.L., Egede L.E. (2012). The Impact of Social Support on Outcomes in Adult Patients with Type 2 Diabetes: A Systematic Review. Curr. Diab. Rep..

[16] Abolfotouh M.A., Al-Khowailed M.S., Suliman W.E., Al-Turaif D.A., Al-Bluwi E., Al-Kahtani H.S. (2012). Quality of life in patients with skin diseases in central Saudi Arabia. IJGM.

[17] Aroian K., Templin T.N., Ramaswamy V. (2010). Adaptation and Psychometric Evaluation of the Multidimensional Scale of Perceived Social Support for Arab Immigrant Women. Health Care Women Int..

[18] Pérez-Villalobos C., Briede-Westermeyer J.C., Schilling-Norman M.J., Contreras-Espinoza S. (2021). Multidimensional scale of perceived social support: Evidence of validity and reliability in a Chilean adaptation for older adults. BMC Geriatr..

[19] Asmri M.A., Almalki M.J., Fitzgerald G., Clark M. (2020). The Public Health Care System and Primary Care Services in Saudi Arabia: A System in Transition. East. Mediterr. Health J..

[20] Alotaibi H.M., Alruwaili Z.M., Dilli A.A., Altaleb A.A., Asiri M.M., Alwadani O.J., Alshaalan Z.M., Dar U.-F. (2023). Assessment of Primary Care Physicians’ Expertise of Common Dermatological Conditions in the Jouf Region, Saudi Arabia: A Mixed Methods Study. Healthcare.

[21] Lovibond S.H., Lovibond P.F. (1995). Manual for the Depression Anxiety & Stress Scales.

[22] Finlay A.Y., Khan G.K. (1994). Dermatology Life Quality Index (DLQI): A simple practical measure for routine clinical use. Clin. Exp. Dermatol..

[23] Zimet G.D., Dahlem N.W., Zimet S.G., Farley G.K. (1988). The Multidimensional Scale of Perceived Social Support. J. Personal. Assess..

[24] Li F., Luo S., Mu W., Li Y., Ye L., Zheng X., Xu B., Ding Y., Ling P., Zhou M. (2021). Effects of sources of social support and resilience on the mental health of different age groups during the COVID-19 pandemic. BMC Psychiatry.

[25] Bewley A. (2017). The neglected psychological aspects of skin disease. BMJ.

[26] Germain N., Augustin M., François C., Legau K., Bogoeva N., Desroches M., Toumi M., Sommer R. (2021). Stigma in visible skin diseases—A literature review and development of a conceptual model. Acad. Dermatol. Venereol..

[27] Jankowiak B., Kowalewska B., Krajewska-Kułak E., Khvorik D.F. (2020). Stigmatization and Quality of Life in Patients with Psoriasis. Dermatol. Ther..

[28] Costeris C., Petridou M., Ioannou Y. (2021). Social Support and Appearance Satisfaction Can Predict Changes in the Psychopathology Levels of Patients with Acne, Psoriasis and Eczema, before Dermatological Treatment and in a Six-Month Follow-up Phase. Psych.

[29] Soman S., Bhat S.M., Latha K.S., Praharaj S.K. (2016). Gender Differences in Perceived Social Support and Stressful Life Events in Depressed Patients. East. Asian Arch. Psychiatry.

[30] Caetano S.C., Silva C.M., Vettore M.V. (2013). Gender differences in the association of perceived social support and social network with self-rated health status among older adults: A population-based study in Brazil. BMC Geriatr..

[31] Tam C.L., Lee T.H., Har W.M., Pook W.L. (2011). Perceived Social Support and Self-Esteem towards Gender Roles: Contributing Factors in Adolescents. Asian Soc. Sci..

[32] Dalgard F.J., Bewley A., Evers A.W., Gieler U., Lien L., Sampogna F., Ständer S., Tomas-Aragones L., Vulink N., Kupfer J. (2018). Stigmatisation and body image impairment in dermatological patients: Protocol for an observational multicentre study in 16 European countries. BMJ Open.

[33] Weinberger N.A., Mrowietz S., Luck-Sikorski C., von Spreckelsen R., John S.M., Sommer R., Augustin M., Mrowietz U. (2021). Effectiveness of a structured short intervention against stigmatisation in chronic visible skin diseases: Results of a controlled trial in future educators. Health Expect..

[34] Ozbay F., Johnson D.C., Dimoulas E., Morgan C.A., Charney D., Southwick S. (2007). Social Support and Resilience to Stress. Psychiatry.

[35] Song Y., Song H.J., Han H.R., Park S.Y., Nam S., Kim M.T. (2012). Unmet Needs for Social Support and Effects on Diabetes Self-care Activities in Korean Americans With Type 2 Diabetes. Diabetes Educ..

[36] Lahousen T., Kupfer J., Gieler U., Hofer A., Linder M., Schut C. (2014). Differences Between Psoriasis Patients and Skin-healthy Controls Concerning Appraisal of Touching, Shame and Disgust. Acta Derm.-Venerol..

[37] Hrehorów E., Salomon J., Matusiak L., Reich A., Szepietowski J.C. (2012). Patients with psoriasis feel stigmatized. Acta Derm.-Venereol..

[38] Zeiser K., Hammel G., Kirchberger I., Traidl-Hoffmann C. (2021). Social and psychosocial effects on atopic eczema symptom severity—A scoping review of observational studies published from 1989 to 2019. J. Eur. Acad. Dermatol. Venereol..

[39] Zhang X.J., Wang A.P., Shi T.Y., Zhang J., Xu H., Wang D.Q., Feng L. (2019). The psychosocial adaptation of patients with skin disease: A scoping review. BMC Public Health.

[40] Dalgard F., Stern R., Lien L., Hauser S. (2012). Itch, stress and self-efficacy among 18-year-old boys and girls: A Norwegian population-based cross-sectional study. Acta Derm.-Venereol..

[41] Yew Y.W., Kuan A.H.Y., Ge L., Yap C.W., Heng B.H. (2020). Psychosocial impact of skin diseases: A population-based study. Kaliyadan F, editor. PLoS ONE.

